# More Than Words Can Say: A Multi-Disciplinary Consideration of the Psychotherapeutic Evaluation and Treatment of Alexithymia

**DOI:** 10.3389/fpsyt.2020.00433

**Published:** 2020-05-25

**Authors:** Patrice Duquette

**Affiliations:** Private Practice, Birmingham, MI, United States

**Keywords:** alexithymia, psychotherapy, interoception, interoceptive inference, mentalization, levels of emotional awareness, TAS-20, predictive processing

## Abstract

Alexithymia is a disorder that stands at the border of mind and body, with psychological/affective and physiological/experiential disturbances. The purpose of this article is to propose a new clinical access point for the evaluation and treatment of the deficits in emotional awareness demonstrated in alexithymia. This will be based on insights from recent neuroscientific research, which is adding to the psychodynamic understanding of alexithymia, regarding clinical presentation and etiology. Following a brief review of definitions, forms of measurement, and potential etiologic elements of alexithymia, current neuroscientific theory and research into “predictive processing” approaches to brain function will be outlined, including how “interoception” and “interoceptive inference” underpins emotion and emotional awareness. From this synergistic perspective, I will outline how interoceptive inference provides a key to the link between: problems in early life relational experiences and the patient’s long held, but suboptimal models of their inner and outer world. This is reflected in the deficits in affective experiencing and emotional awareness described in alexithymia. Three clinical cases will be presented to illustrate this nuanced consideration of alexithymic etiology and treatment. The implications of the historical, psychological, and somatic aspects of experience will be considered, regarding the patients’ diminished ability to: experience and represent emotional experience as distinct feeling states; signify the relevant meaning of affective experience; and incorporate such with cognitions to adaptively guide behavior. These will be addressed using psychometric, psychological, neuro-cognitive, and neurocomputational approaches. Elements from current theory, research, and treatment of alexithymia, will be highlighted that are salient to the clinician, in order to support their understanding of patients against the backdrop of current psychodynamic and neuroscientific research, which will thereby increase treatment options and benefits. The focus, and conclusion, of this article is the role that attention to interoception can play (within the safety of the therapeutic relationship and within any therapeutic process) in allowing updating of the patient’s strongly held but dysfunctional beliefs.

## Introduction

The construct of alexithymia has been studied from many different clinical and neuroscientific perspectives. It was originally organized around common clinical characteristics that psychosomatic patients present, notably their difficulty in verbalizing their emotions as feelings. The word *alexithymia*’s Greek roots describes this lack of the language for feelings (1, 2). Subsequently, the concept has taken on more depth and meaning, now denoting a disturbance in: the individual’s affective experience; level of emotional awareness; and, ultimately, verbalizations of emotions as feeling states. There has, however, been persistent difficulty across disciplines in creating a comprehensive clinical and neurobiological account of the disturbance and in representing this in adequately descriptive language that is understandable by all disciplines and which takes account of the inherent variability in clinical presentations of alexithymic patients. As a clinician, it is my intention here to introduce therapists to research and theoretical propositions that are relevant to psychological and neuroscientific perspectives regarding alexithymia.

Following a brief review of the history of the concept of “alexithymia,” I will address measurement constructs and review proposals regarding psychological approaches to etiology, focusing on what part early-life development may play in the emotional, relational and bodily-oriented aspects of the disturbance. Neurobiologic theory and processes, that provide insight into both etiological and clinical considerations, will be presented in some detail, to facilitate the clinician’s understanding. The relevant neuroscientific terms and theory will be explained, where required.

As a clinician, it is my intention to present a unique perspective of alexithymia, bringing together new information from clinical and scientific disciplines. This will involve contextualizing current psychometric measurement and psychological theories of alexithymia within current neuroscientific theory of how “predictive processes” create emotional experience. These processes rely on: physiological necessities, prior beliefs, and sensory evidence from the environment (in this paper, the focus is on bodily experience). I will link psychological and neuroscientific proposals to explain how an alexithymic patient’s unique symptomatic presentation is generated and how this may be best comprehended and responded to in their treatment.

## History of the Definition and Measurement of the Alexithymia Construct

The concept of alexithymia arose from the clinical presentation of symptoms, noticed by a small number of clinicians, who were impressed by distinct similarities in how these patients expressed their emotional state as feelings ^1^, or, more accurately, could not describe their emotional states. Focus was initially on patients with psychosomatic illnesses, whose thought processes were seen as *“utilitarian”* and concrete, with apparent minimal ability to fantasize or abstract. This form of thought was labeled *“pensee operatoire*” (4) because patients’ vocabulary for their emotional states often utilized only somatic descriptions, focusing on external events as causative for internal experiences. However, patients also exhibited behaviors that were disorganized and appeared to be affectively charged, such as poor impulse control and chaotic relationships—expressing no recognition of the affect evidenced in their behaviors (2). Clinicians proposed that such patients did not recognize their emotions through symbolic representation, nor perceive them as signals regarding their needs or wants (5), but rather enacted their feelings in behavior, or referenced affective experience through somatic sensations or somatization.

A consensus was reached on the definition of alexithymia at a 1977 conference on psychosomatic research in Heidelberg, Germany. The definition was organized around the clinically salient features: 1.) difficulty identifying feelings and in distinguishing between feelings and bodily sensations that reference emotions; 2.) difficulty describing feelings to others; 3.) restricted imagination and lack of fantasy; and 4.) an externally oriented thinking style (6–8). Subsequent research has proven alexithymia to be a multi-faceted dimensional personality trait, with varying degrees of expression in individuals (9–11), including individuals who have diagnosable psychiatric illness but also healthy individuals (12). Clinically relevant alexithymia is thought to affect 10% of the general population (13, 14).

Alexithymia is a complex construct that involves both cognitive and affective disturbances. It is considered a deficit in experiencing, describing, and regulating emotion (8), evidenced as a more global deficit in emotional awareness, involving an inability to bring emotional experiences to the level of conceptualizing feelings regarding oneself or others (15). Although there is consensus on the factors comprising alexithymia, there is much disagreement over how to test and quantify these. The most common measurement approaches will be addressed below. (For a more extensive review the reader is directed to Lumley et al. (16) and Maroti et al. (17).

The Toronto Alexithymia Scale (TAS), which is the most commonly used self-report measure, was originally developed as 26 items and then pared down to a 20-item questionnaire (TAS-20). The TAS-20 provides individual sub-scores for the three factors from the Heidelberg consensus i.e.: 1.) Difficulty Describing Feelings (DDF); 2.) Difficulty Identifying Feelings (DIF); and 3.) Externally Oriented Thinking (EOT). Daydreaming, as a potential factor, was eliminated from the TAS-20, as it was considered subject to social responsivity bias and insufficiently reflective of the subject’s capacity for imagination (8). The overall score generated by the TAS-20 is intended to indicate severity of alexithymia generally. This is widely used within research settings, with cutoff scores indicating the presence (greater than 61) or absence (under 51) of clinical levels of alexithymia. Advantages of the TAS-20 are that it easy to use, brief and has good construct validity (9) and reliability (17).

By contrast, the Levels of Emotional Awareness Scale (LEAS), developed by Lane and co-workers, reflects their conceptualization of alexithymia as a global impairment in affective processing, resulting in deficits in emotion generation, description and awareness (18). The LEAS requires written answers to questions that are intended to elicit feelings—within the subject and in their relation to others. The test rubric allots points for the degree of differentiation in the emotional words used, as well as in the differentiation of the self from other. Therefore, if the individual uses more specifically affectively laden language, and/or describes blends of feelings, and language that differentiates self from other, they will receive a higher score. The LEAS has good inter-rater reliability, internal consistency, and criterion validity (17). A strength is that only the structure of experience is addressed, not the content, which limits the subject’s ability to falsely enhance their scores in order to make a desirable impression. The LEAS is linked to the construct of Emotional Awareness (15, 19, 20) as well as to the Three Process Model (TPM) of emotional experience (21–24).

The TAS-20 and the LEAS are both commonly used throughout research and in clinical literature and practice. Importantly, the creators of both measurement tools agree that a disturbance in emotional processing is salient to alexithymia (9, 16). However, both measures have been criticized. It is now recognized that they potentially measure different aspects of alexithymia.

Furthermore, as Lumley notes, the TAS-20 was the first readily reproducible test and has been very commonly used because it is simple to administer. As a result, it has become, by default, improperly considered actually to *define* alexithymia. However, *“the measure one is using, (e.g., TAS-20, OAS, BVAQ, LEAS, etc.) is not itself alexithymia but only an approximation of it”* [(25), p. 241]. We must be mindful, when considering the results of any particular measurement instrument, that alexithymia is *not* a measured score on any given test but, is the affective and cognitive difficulties that characteristically present in some patients. In this article, I will, therefore, treat the TAS-20 as a descriptive measure of predominant deficits that present clinically in an alexithymic patient. I will also address the construct of Emotional Awareness but again as a descriptive measure, not as an empirically measured value.

## Psychodynamic Consideration of Alexithymia

Psychoanalytic theorists have discussed alexithymia from different perspectives for decades [e.g., (5, 7, 26)]. Krystal (5) hypothesizes that alexithymia could result from: developmental disruptions in affect development related to infantile psychic trauma; post-traumatic disturbances due to trauma experienced later in life (particularly if anhedonia is a significant component of the presentation); and/or neurobiologic dysfunction. In his clinical opinion, alexithymic symptoms are most often associated with *“impairment in the capacity for self-care”* [(5), p. 243)]. Moreover, he posits that patients are fundamentally unable to recognize and utilize emotions as signals to themselves. They can’t realize their emotions as feelings, nor can they recognize them as vital and necessary reaction to their lived experience. He elegantly describes this underlying difficulty.

*“Only when one experiences the cognitive aspect of an emotion—the meaning of the affect and some indication of the “story behind it”—and simultaneously has the expressive reaction and an adequate capacity for reflective selfawareness [sic], can one observe that one is experiencing a ‘feeling’ and identify it.” [(5), p. 243*]

According to Krystal’s interpretation, the cognitive disturbances of alexithymia represent a lack of affective vitality, as evidenced in the content of verbalizations, which are externally oriented and monotonous. They may seem well adjusted to reality, *“superadjusted”* in fact (5). However, this present-minded focus overlies a severe paucity of imaginative capacity. Thought is stimulus-bound, with utilitarian attachments and a significant lack of relational involvement with others.

Other theorists highlight that the results of this outward focus is to leave the individual with bodily sensations that are confusing and can trigger somatic disturbance which increase somatization, without the means to break out of this cycle through language and relationship (8). Such a lack of resonance and cohesion between bodily sensation and awareness of their emotional implications leaves alexithymic patients with a distinct inability to symbolize and therefore to regulate their emotional states, leaving them vulnerable to dysregulation, both somatically and psychologically. The functional capacity of *“mentalized affectivity”* (27) is lacking, such that they are unable to: identify; regulate; or communicate their emotional state to themselves or others; and therefore unable to utilize relationships to emotionally regulate (28). As we shall discuss below, reigniting emotional experience and awareness is an important element of psychotherapy with alexithymic patients, within any theoretical formulation.

Regarding patients with both alexithymia *and* anhedonia, Krystal (5) asserts that this combination is reflective of *later* (rather than early) life trauma that can create an experience in which the trauma continues to be subjectively experienced, as if it were punishment for the patient’s transgression in seeking their own goals. Thus, reaching for any goal generates distress, with the trauma implicitly acting as a command to the individual not to experience positivity or vitality. McDougall’s (26) noted that anhedonic alexithymic patients have a significant distortion in their self-representation, which does not allow them to even *“be”* themselves, much less to take care of their needs (26). They have diminished access to all affective experience, so as not to touch any pleasurable emotion, with the anhedonia resulting from an inability to play, to demonstrate pleasure, or to seek well-being.

Using Piaget’s theory of cognitive development as a template, Lane and Schwartz (15) developed a concept they refer to as Emotional Awareness, which is directly applicable to the deficits in alexithymia. This construct provides a means to: consider the experiential aspect of emotion; provide support to clinical exploration of individual differences in emotional experience: determine, and potentially measure, how this might change over time (15). In their formulation, Emotional Awareness is a trait that represents a cognitive *ability*, or skill, developing in a step-wise progression over time. Thus, an individual’s ability to detect and describe their emotional experience, and that of others, undergoes structural transformation, with increasing differentiation. There is a progression from (implicit) bodily-based sensory experience through levels of cognitive awareness that are increasingly more explicitly and consciously recognized. Alexithymia represents dysfunction, with the patient operating at a Level of Emotional Awareness that is limited by specific developmental disturbances that have created deficiency in some of the elements necessary for emotional awareness at higher levels. For example, there may be deficits in awareness of bodily sensation or in recognition of emotions as feeling states. Although this progress represents a continuum of a trait-level ability, Emotional Awareness is conceived as having five levels. (For further information the reader is directed to (15, 19).

Level 1: Awareness of bodily sensations: the individual’s experience of emotion will only be reflected in a report of the sensations from their body

Level 2: Awareness of the body in action: emotional experience is through action tendencies and sensation; the ability to experience emotion does not reach the level of feeling

Level 3: Awareness of feeling states: the quality of emotional experience changes to include somatic experience as well as psychologic experience

Level 4: Awareness of blends of feelings: there is an increased range of emotional awareness and this is more coherent and can be recognized to change over time; yet the recognition of other’s emotional states is still unidimensional

Level 5: Awareness of blends of blends of feelings: there is greater capacity to recognize and make distinctions in emotions, with descriptions more nuanced and linguistically complex; and the ability to differentiate self from other is most available

Lane and colleagues have incorporated into their model of Emotional Awareness current neuro-cognitive and neuro-computational theories to proposes how different elements of affective processing *“interact to produce self-reportable emotional experience”* [(29), p. 474]. They call this the Three Process Model (24, 30, 31) and assert that disturbances in the elements of affective experience, described within this model (which may be created by various mechanisms that are dependent on the individual’s life history), are reflected in lower Levels of Emotional Awareness. First, however, in order to allow deeper insight and understanding by clinicians, it is necessary to review the recent neuroscientific concepts and theories that underpin such models.

## Overview of Related Neuroscientific Concepts and Research Findings

### Interoception, Homeostasis, and Allostasis

Neuroscience is generating increasing research into how the synthesis of bodily sensation with other sensory input (as well as top-down influences) creates emotion and feelings. Fundamental to this is “interoception,” which is the set of senses that sends information from all the tissues of the body to the brain, through neural and blood-borne pathways (32), being formally defined as the *“the process by which the nervous system senses, interprets, and integrates signals originating from within the body, providing a moment-by-moment mapping of the body’s internal landscape across conscious and unconscious levels”* (33).

The insular cortex is an area that has widespread importance regarding interoception and emotional experience (34, 35), with interoceptive signals primarily entering the posterior aspect, and the anterior aspect more important relative to the multitude of connections that support subjective experience. The anterior insula cortex (AIC) and the Anterior Cingulate Cortex (ACC) play important complementary roles regarding experience and behavior, serving as interdependent arms of a coordinated system (32). The anterior insula is functionally important towards the generation of feeling states and subjective awareness, while the anterior cingulate is related more to motivation and behavior (34, 36).

In addition to the coordinated activity between the AIC and the ACC, each is also involved in activity with other areas that function together more generally as networks. The resulting connectivity of these networks generates specific functional activities which are dependent on activity within and between linked areas. It has been proposed that the Default Mode Network^2^ (DMN), *“is in charge of processing self-related content”* [(38), p. 55]. This network is most active when the brain is “idling.” Fluctuation in its activity are thought to be related to the degree to which individuals are thinking introspectively (39). Utilizing fMRI, Liemburg and colleagues (40) found different levels of activity in alexithymic subjects when compared to healthy control subjects. Notably, there was lower connectivity in the anterior area of the DMN that is thought to be most active in emotional processing. They attribute this to the decreased emotional awareness in alexithymia (40, 41).

Crucially for our discussion, it is now understood that the subjective experience and awareness of emotion relies on interoceptive signals from visceral, autonomic, hormonal, and immunological systems that indicate bodily changes [e.g., (32–34, 42–44)]. It is generally accepted that emotion involves the integration of interoceptive sensations (which provide information about the current state of the body) together with exteroceptive information from the external world (which contextualizes how the state of the world is likely to impact on the state of the body). This integration is performed by top-down brain processes that involve information (i.e. what is known or understood) about the person’s past expectations and their understanding of the probable affordances, or possibilities for action within the present environment (45). We will consider the aspects of physiological process for which interoception forms the foundation, before addressing how the deficits noted in alexithymia can be considered in this light.

The continuous flow of interoceptive signal from the body to the brain necessarily provides information about the homeostatic state of the body (which is mostly unconscious). Homeostasis is conceived as a reactive process that is constantly countering an inherent biologic instability within the organism (46).

A more useful characterization of bodily stability is allostasis [(47), p. 1192], which is the process by which the organism prospectively avoids disruptions to homeostatic set points (47–50). Interoceptive information is utilized by allostatic processes to control bodily states by anticipating perturbations (such as energy demands) and implementing allostatic mechanisms within the internal milieu, through ANS reaction or motoric actions within the environment, heading off potential homeostatic changes *before* they actually occur (51). Interoceptive sensations, therefore, are signals to the brain regarding the motivational state of the individual, with ANS activity acting as the effector arm of this processing (32). Thus, the interoceptive signals that underpin homeostasis and allostasis play an essential role in motivated behavior (34, 52, 53). For example, interoceptive signals that indicate falling body temperature will motivate an organism to search for warmth and shelter *before* any vital homeostatic set point is breached. Importantly for this paper, significant allostatic needs are always present, in some form, in a person’s social environment, and must be responded to (50)^3^.

To better comprehend how sensations interact with top-down influences to create emotional experience and awareness in healthy individuals, as well as those with alexithymia, we must follow a path through neuroscientific theory and research that appears complex at first. However, if the reader contextualizes this information with some personal reflection from their daily experience, it will become increasingly understandable.

## Computational Neuroscience: Prediction, Probability, and Inference

Recently, computational neuroscience and the use of mathematical modelling has suggested a radical way to understand brain processes. The utility of this approach has great potential in psychiatry and psychosomatic medicine (57, 58, 59).

Crucially, we start from the fact that the human brain does not have direct access to the world. Therefore, in order to make sense of its internal environment (its own body) and the external environment (the world it inhabits), it has to *infer* the causes of the sensory signals it receives from the inner and outer world, in order to decide how best to react, in order to enhance the organism’s chances of survival. Predictive processing theory starts from this point and asserts that our brains use our previous experience as the basis for “predicting” (i.e. inferring or hypothesizing) what is the most likely cause (i.e., meaning) of incoming sensations from both our internal and external worlds (60–62). This inferential activity produces what are called “generative models,” *“which attempts to predict each wave of sensory input before it arrives”* [(24), p. 30]. Our brain builds these models, throughout our lives, and continuously modifies them in the light of incoming sensory signals, hopefully so that they provide increasingly accurate approximations of the reality in which the individual finds themselves, at any given moment in time, in order to guide behavior. In other words, we learn and adapt by continually updating our generative models for what are the most likely causes of any particular set of incoming sensory information.

Predictive processing describes this model-building in the brain by using Bayes’ Theorem, to illustrate how our generative models (also sometimes—confusingly—called hypotheses, predictions, priors, expectations, or beliefs) are built and then tested against the incoming interoceptive and exteroceptive sensory streams. Frith (63) explains Bayes’ Theorem thus: *“Given some phenomenon (A) that we want to know about, and an observation (X) that is evidence relating to A, Bayes’ Theorem tells how much we should update our knowledge of A, given the new evidence X”* [(63), p. 121]. If we assume that the brain behaves as a “Bayesian observer,” we can assume that it is constantly attempting to “*know about*” the hidden causes of its internal and external sensations (i.e. phenomenon A, using incoming sensory data X). Previously learned predictions (or priors) are thus continually being evaluated against the incoming flow of sensation (i.e. observation X) and being potentially updated. Prediction, in this context, is a technical term, not the cognitive “act” of predicting. It is a, generally unconscious, quantitative, and probabilistic attempt to match best examples from previous learning to what the sensory organs are currently receiving. Bayesian inference thus allows the brain to predict (i.e. hypothesize) what is the mostly likely cause of a given (set of) sensation (whether coming from within the body or from the outside world).

This Bayesian process occurs throughout the brain, in a step-wise manner. Thus, the generative models it creates are termed hierarchical. Predictions (i.e. our prior beliefs or expectations) essentially flow down the hierarchy, meeting upcoming sensation from interoceptive, exteroceptive, and proprioceptive sources. When any given prior prediction (the likelihood that sensory inputs would be observed under a given model) is compared against sensory data, further activity in the hierarchical processes determines whether to update that prediction in the light of this new, incoming evidence. To do so it utilizes the difference between the prediction and the sensory evidence. This difference is referred to as “prediction error”—i.e., how wrong the prediction appears to be, when compared to the current sensory data. This hierarchical activity is occurring across multiple levels of the brain at any given instant. Whether any prediction error will update a prior prediction, or whether actions will be generated, following the sensory evidence, depends on the relative salience/reliability of the prior prediction compared with that of the sensation.

To understand this, it is important to reiterate that the words expectation, prior, or belief, do not denote consciously held beliefs, but refer to activity occurring in neurons in the brain. Referring back to our Bayes’ example above: all priors (beliefs regarding phenomenon A); incoming sensory information (observation X); prediction errors generated (if phenomenon A doesn’t match X); and resulting posterior beliefs (updated beliefs about phenomenon A), are assumed to be held in the form of probability distributions. There is, necessarily, uncertainty associated with each of these probability distributions, reflected mathematically by its relative spread. The inverse of this spread is known as its “precision,” which denotes the salience (confidence or reliability) associated with a particular prior or prediction error (64). The precision (salience) imparted to the prior/prediction and also to the incoming sensation is modulated by many factors (e.g., neurochemical, motivational, strength of sensory signal). Crucially, this salience is used by the brain to weight the priors *vs.* prediction errors. The relative weight (i.e. precision) of the prior belief *vs.* the incoming interoceptive sensory information, determines whether the prior gets updated (or not). If the prior is precise, it will tend not to update in the light of imprecise prediction error. By contrast, highly precise incoming sensation can update an imprecise prior. Updating results in a new (hopefully more accurate) belief known as the “posterior” belief. Such updating of beliefs/predictions occurs across all biopsychological domains, including: perception; motor control; emotion; and decision-making.

Predictive processing has been applied within a wider theory of adaptive functioning known as the “Free Energy Principle” (61, 64). This principle asserts that organisms’ adaptive changes are *always* in the service of minimizing unexpected (or unpredicted) sensations. Failure to do so will tend to lead to dis-homeostasis, with increasing disorder (entropy) that is dangerous to the organism, ultimately likely to cause death. Through the process of Bayesian updating, therefore, the organism is constantly improving its ongoing modeling of its inner and outer sensory world. By minimizing unexpected sensations, it maintains the body in stable homeostatic/physiologic states. Human beings thus form and update predictions (beliefs or expectations), throughout our lives, about all aspects of our inner and outer worlds. For example, we have prior beliefs/predictions about: homeostatic set points; the meaning of a pain in our body; and our social experience regarding the intentions of another person in the world.

It is important to note that our beliefs (priors), and thus our (generative) models of the world and ourselves in it, can never be veridical but they must be good enough to allow us to survive.

The power of the Free Energy Principal is that it goes beyond providing an account of how the brain adjusts its perceptions (61). This Principal also explains how actions are generated by a process known as active inference (61, 65, 66). In this perspective, motor reflexes are enslaved by predictions about the results of the movement. For example, a generative model of movement involves a prediction that a particular set of muscles will contract in a particular way to produce a particular set of sensations from interoceptive, proprioceptive, and exteroceptive sources, with the prediction errors resolved only if the muscles comply with these predictions to bring the actual sensations in line with the predicted sensations. It is important to note that an “action” taken for the purpose of minimizing prediction error in a generative model can be from: the major muscle groups of the body; the viscera or internal organs of the body; and also through actions of the autonomic nervous system.

Significantly for our purposes, active inference processes are thus also relevant to interoception, homeostasis, and allostasis. The homeostatic set point is itself a prediction. If one considers that allostatic stability is an absolute necessity for the human organism, the brain must constantly be one step ahead of what the incoming sensation implies regarding *future* homeostasis, using its prior predictions based on our past experience to do so. Any deviation from such homeostatic set points produce prediction errors will actively engage allostatic responses to maintain energy regulation (49). Some examples are, visceromotor reactions such as autonomic reflexes (e.g., heart rate, vasodilation, release of hormones), or behavioral actions (reaching for food or water), thus creating a new posterior prediction and continuing the generative process [e.g., (67)]. The hierarchical belief updating that refers specifically to interoceptive signals from the body is known as interoceptive inference (68).^4^

A further aspect of this process is that, ideally, the individual should regularly be seeking further evidence for its models, through its own action or perception, in order to check that its models of the world (and body) are reasonably fit for purpose, thus resolving present and future uncertainty. This seeking is termed “*epistemic foraging*” (61). An important corollary is that epistemic foraging actions will only occur during times when the individual perceives the environment as safe; otherwise its energy will be directed entirely toward protective activity, with no remaining energy available for seeking out new data.

## The Body in Interoceptive Inference

In light of the theories outlined above, we can now bring together the theoretical perspectives that indicate the crucial importance of the body in the interoceptive inference process that is responsible for generating elemental aspects of experience, such as emotional experience, emotional awareness, and a sense of self.

As described above, interoceptive inference processes occur through a constant hierarchical monitoring of changes in bodily state. Their effect on interoceptive predictions, and subsequent adjustment of homeostatic and allostatic processes, is what engenders emotional content (49, 68, 69). The insular cortex is a vital hub regarding emotion, because interoceptive sensations from the body and predictions about the world, as well as from “higher” brain areas are integrated here. This *“primary interoceptive cortex”* (67) is thus a clearing house, comparing predictions against afferent interoceptive information. As beliefs, or priors, are updated, emotions are consequently generated (49, 69). Further iterations of interoceptive inference of emotional state will ultimately lead to emotional awareness (70).

Moreover, the Free Energy Principle implies that the highest priority will be given to the self-regulating mechanisms of the body, i.e. to homeostatic and allostatic processes that maintain survival (71). Allen and Tsakiris claim that this privileged status, which is a function of the necessity for the body to maintain stability, dictates that the homeostatic functions of the body are, in their words, the *“first prior*.*”* In other words, the salience of any sensory stimulus will always first be evaluated in relation to the impact it will have on regulatory survival processes.

Finally, the result of this high precision afforded to visceral sensations allows visceral sensations to become a consistent *“‘anchor’ on which to lodge a more permanent feeling of bodily self”* [(71), p. 41]. Thus, visceral sensations provide an implicit sense of self-awareness that may not always be available to conscious access but, pertinently, may still be an important influence in the hierarchical modeling process. Notably, for our patients with alexithymia, the balance between the relative strength (the precision) of prior beliefs *vs.* interoceptive information is elemental to aspects of health and psychopathology regarding emotional experience and awareness.

## Factors in the Early Origins of the Interoceptive Inference Process

Infancy is recognized as a vital time in the formation of links between interoceptive processes and experience. Attuned caretakers are an absolute necessity to maximize the emerging capabilities of the infant. How the child’s and adult’s emotional life will unfold depends on how this early vital inference process occurs (72). Close repetitive embodied interactions act as homeostatic regulators for the infant, and the quality of that interaction amidst the origins of inferential processing is the basis for the development of early expectations, or priors, about the causes of our sensory states. Furthermore, these early interactive physical activities stimulate the development of *“embodied mentalization”* in the infant which is the basis for an early sense of self and directly impacts the ability of the growing child to develop their own models of their interoceptive states and subsequent adaptive strategies for free energy minimization (72).

The import of relationships in infancy in developing beliefs about our body and sense of self is highly applicable to alexithymia. For example, if there is a sudden unexpected change in an infant’s somatic sensation and this is not resolved within the consistent presence of a caretaker, the effect of the “surprising” interoceptive sensations in the resulting hierarchical inference processes is magnified, and may be negatively compounded over time. If cognition is as much affected through embodied experience as Allen and Tsakiris (71) assert, then these unsupported changes in the infant’s embodied perception of the world will also have important effects on future cognitions. With very limited resources available to the infant who is experiencing emotion (and who has no ability to comprehend cause and effect), life is a constant surprise. One can see how important the original caretaking relationship is for an individual’s tendency to expect safety in the world and thus for their ability and willingness to engage in epistemic foraging. Attuned caretakers are an absolute necessity to maximize the emerging capabilities of the infant. How the child’s and adult’s emotional life will unfold depends on how this early vital inference process occurs (72).

The power of such experiences can be imagined in the example of an individual who suddenly experiences a strong sense of danger to their bodily state. This is often experienced as a change in visceral sensation (muscles tensing quickly, a sense of distress in the gut), which gives rise to sudden highly precise interoceptive prediction errors, which then impact on generative models, activating priors unique to each individual in that present moment experience. Exteroceptive contextual elements, embedded in this experience, may also stimulate highly precise visceral predictions about what these sensations mean, generalizing to the whole body’s response. While in adult life an individual may be able to have cognitive/verbal associations to such physical sensations, and comprehend/express that they feel fear, there are innumerable events in infancy that are experienced as immediately unsafe, *per se*, suddenly overwhelming the infant’s physiologic homeostatic processes, with such sensory experience entirely pre-verbal. Such experiences, and the infant’s caretakers response to such experiences, as interactions between the body and the world (and other bodies), are the origins of the generative models (predictions/priors/beliefs) and access to interoceptive information (sensation/awareness), that we carry throughout life, about the safety of our body/ourselves in the world.

As noted in psychological theories, it follows that if the alexithymic individual cannot generally recognize bodily signals (for whatever initial reason in early life or through later trauma), bodily sensations that do manage to breach the level of conscious sensation and are experienced, are more likely to be felt as surprising than by those people who are not alexithymic. The effect on cognitive processes is likely be evidenced in the symptoms described as Difficulty Identifying Feelings (DIF), and Difficulty Differentiating Feelings (DDF), as such “surprise” will readily disrupt the coherence of a narrative focus of experience, and therefore any verbal ability to express emotion as feeling states, affecting the individual’s level of emotional awareness. Moreover, if this surprising sensation is hierarchically evaluated by a prior for physical illness or frailty, that happens to have high precision, the expression of the experience would be somatically oriented. Indeed, the lack of embodied sensation as an *“anchor”* would support the alexithymic individual’s tendency to focus on the external world, in order to create an external anchor, leading to externally oriented thinking.

## Emotional Awareness and the Three Process Model

Crucial to alexithymia is the question of how best to characterize emotional awareness. Smith, Lane, and colleagues’ solution is their Three Process Model (TPM) (24, 31, 45) which incorporates interoceptive inference, learning and brain processes, to propose how *“an emotion episode is initiated”* within the individual, in response to events that are *“real, remembered, or imagined”* [(24), p. 35]. The Three Process Model breaks down the elements of emotional experience into three factors (see below), which depend on predictive processing, neural network, and global workspace theories of brain functioning (24, 31). The authors create a cohesive model of how different aspects of: the initial response to the event; the bodily sensation; cognitions; motivated behavior; and the recognition of the response that fits an emotion-concept category (e.g., fear, sadness, etc.) may all ultimately become available to conscious awareness. The three processes (steps) designated by the model are (24, 31, 45):

1. Affect Response Generation (ARG): somatovisceral and cognitive processes are modulated through interoceptive inference, in a context-dependent appraisal of the salience of a real or imagined stimulus (which is often implicit), based on for example: the metabolic and behavioral demands; goal-achievement needs regarding elements such as the expectedness/novelty, or a sense of agency related to the event.
2. Affect Response Representation (ARR): the somatovisceral component of the affective response that has been generated is *“perceived via afferent sensory processing”* [(45), p. 5] and is then updated and conceptualized as a certain feeling category that describes the current emotional experience (e.g. fear, anger, etc.).
3. Conscious Access (CA): a process that grants access to domain general cognition for somatovisceral perceptions and emotion concepts, and are thus available for verbal expression and voluntary emotional regulation strategies.

The TPM allows a thorough evaluation of each patient’s symptoms and their individualistic expression of alexithymia. In their computationally modeled clinical presentations, Lane and colleagues are able to account for the differences in symptomatology evidenced in alexithymic patients, with a view to improving individualized treatment approaches (24, 29, 30, 45). There is a strong emphasis on the importance of prior experience (and thus on predictions/expectations regarding internal and external states) and also on the inferential process of any emotional experience (24). If there is disturbance in any element of the TPM, the resulting Level of Emotional Awareness will be affected. For example, if a person is limited in their ability to appraise a situation, due to strong priors that quickly “cement” their expectations, the ARG would be circumscribed and they would have a limited range of emotional experience. If there were meager sensory signals available, for reasons associated with trauma and associated somatic disturbance that persists, then emotion concept labeling would result in emotion not being represented (ARR). Finally, a paucity of emotional language could be an indicator that other domain-general processes have created disturbances in how the networks necessary for conscious access (CA) co-operate. (The interested reader is directed to their research that models the influence of the multitude of interactive components of internal state and contextual processes [e.g., 22, 24, 30, 68]).

## The Intersection of Theory and Clinical Presentation

As the Three Process Model assumes, each individual has a personal history that is vital to the expression of their symptoms and their available health. Nemiah (73) stresses that we must allow the individual to tell us their life story and their illness in their words, as they inevitably will put their illness within the context of that story, thus, *“The proper study of psychiatry, is biography”* [(73), p. 460]. While the collection of data and neurobiological etiology is fundamental to psychiatric research and practice, we should always address the patient as a unique individual who develops within relationships (73).

By way of illustration, I now present several clinical vignettes in order to consider alexithymia through an examination of how the theories presented above intersect at the clinical level. Each clinical example will be outlined regarding how the individual’s history, symptomatic presentation, and clinical interaction best evidences: 1.) psychometric evaluation, 2.) psychological dynamics, 3.) Level of Emotional Awareness, and 4.) the Three Process Model.

The patients described here were treated in psychodynamically-oriented psychotherapy, comprising one individual and one group session per week. Attention in the sessions is paid to the patient’s feeling states, with every attempt made to allow full expression while maintaining a self-observant posture. The patient is encouraged to contextualize their emotional experience within the relationships they have had in their past and those that they are currently involved in. The therapeutic alliance is considered to anchor the patient within the process, through real relationship aspects associated with the therapists. Among other relevant goals, therapeutic intentions are to improve: the emotional awareness of the patient; their functional cognitive abilities; and the effective contribution of prior beliefs and feelings to their perspective of themselves and their relationships in the present moment.

There is an initial psychiatric diagnostic evaluation by the therapist/psychiatrist (PD), with an absolute exclusionary diagnosis for treatment, which includes psychosis. The focus of the therapist is directed toward the psychodynamic presentation of the patient, and the links to early relationships and events. The patient is told at the outset of therapy that there is a commitment required of them during the therapy that they not act on feelings, but rather make every attempt to explore, experience, and verbalize them in the therapeutic process. Their individual therapist (PD) is in the weekly group session, together with a co-therapist. The groups are on-going and process oriented, with the members remaining in the same groups throughout their treatment. Treatment length varies.

Each patient gave informed, written consent to be included in this paper. Various aspects of patients’ history and experience have been changed, to protect their identities.

Box 1 **Clinical Case Presentation.**

The most common topic of conversation in Jill’s therapy is the state of her body. She has descriptive words for the most minute change in its physical sensations but very few for her feeling states. When Jill speaks, her eyes dart around the room evaluating everyone’s response. She will hesitate and change course, in reaction to perceived annoyance or disapproval from others, often making self-deprecating comments. When asked how she feels emotionally, she responds in physical description terms, focusing on her heart rate, fullness in her abdomen, fatigue, and feeling like her thoughts are *“cloudy,”* before concluding that she feels anxious. She believes that her body’s strong sensations are indicative of a stress response which will take a physical toll, and that as a result she will get a severe illness or die young. When she began therapy she was encouraged to get a thorough medical evaluation. She was identified as having Irritable Bowel Syndrome and put on a simple regimen by her gastroenterologist. She regularly follows up with her primary care doctor and has been found to have no organic findings in any organ system on multiple screening exams.

Jill has two siblings, one younger by 17 months and the other by 3 years. A prominent memory that Jill reported was of being in a double stroller at about the age of 3 or 4, sitting behind her younger sibling, her mother talking to the sibling and handing him something. This was one of the only early memories she reported originally in her therapy. She didn’t recall saying anything but she was able to associate this memory with a sense of her body being drained of energy, as she watched her mother and sibling interact. She reported that her mother was kind and *“tried to help me,”* but she remembers her mother as very anxious and just *“smoothing over”* the worries that Jill voiced about her friendships at school with simple responses that didn’t feel comforting. If Jill did complain of feeling sick, she remembers her mother would let her stay home from any event.

In one group session, after the therapist had been engaged with another patient, Jill spoke with insight, noting compassionately to the patient that while she could empathize with her feeling response, she thought the therapist was making an important point that she would do well to consider. Jill spoke with clarity, then blushed and ended her response with, *“Oh, I just don’t know, I’m just all confused and feel more nauseated.”* When her therapist asked how she felt emotionally at that moment, Jill blushed and claimed loudly, *“I can’t feel anything else, especially when I get like this.”* When asked what *“like this”* means, she fluttered her hands in front of her abdomen, as if to describe motion moving up her abdomen towards her neck, and said that her heart *“is racing and feels flippy.”* She then became quiet and didn’t speak for the rest of the group session.

In her next individual session, Jill was asked about the interaction, and why she had become so quiet in the group after that interaction. She claimed that she didn’t know why, but it seemed like *“my body just flared up and that was all I could think of and I didn’t want to keep on going on about that.”* She then began listing other stressors in her life, claiming that she *“just makes everything a mess.”* Her therapist remarked that she had heard her speak clearly and kindly to the other patient in the group, and that didn’t sound like a *“mess.”* She blushed and said, *“you’re just trying to reassure me, like it was in my family, just reassurance, but I was always the one who was treated like the ‘sickly one.’”* The therapist asked her how it was that she would have been “the sickly one.” Jill then reported memories in detail that she had only alluded to in the past.

Jill described a trip when she was 12 to visit family in another state. Her father was dropping them off and returning back home to work. The families were in a restaurant, all the adults talking, and Jill remembered feeling like she was going to choke and maybe die, since because everyone was busy talking they wouldn’t see her choking. She did stop choking, and didn’t say anything at the table. A few days later, Jill felt light-headed, nauseous, and as if *“my heart was just racing out of my chest.”* She told her mother, who took her to the emergency room, where there were nothing physical found to explain her symptoms. Her father was called to return early and take the family home. She could only describe feeling *“just upset”* and notes that this was her earliest memory of experiencing strong physical symptoms in the presence of others and withdrawing from them and ruminating on her fears.

The therapist was aware that Jill had spoken for some time without stopping herself to make some self-denigrating comment or to focus on her bodily sensations. She offered this observation to Jill, who looked at her and then quickly looked away.

*“Why did you look away so quickly?”* the therapist asked.

Jill glanced back and said, *“It seems ‘icky.’*”

The therapist paused a minute, then asked *“What does ‘icky’ mean, is that a thought or a feeling?”*

“Sort of both, maybe I’m afraid you’re going to leave because I am “icky”?”

“Well, I am still here, but I realize when you look away, it is as if I am gone to you because you can’t see me.”

*“That’s true,”* Jill said, and slowly but then purposely brought her eye contact to meet the therapist’s eyes.

“What do you make of telling me these stories now? I don’t remember you ever telling me them before.”

*“Something that was said in the group, about how I always imagine the negative. I remember those times as the first times I felt all this in my body, and how much I was focused on how all those sensations felt so bad, but nobody asked me how I was feeling really, not feelings, and all I had was how my body felt to tell me anything, and that left me just feeling ‘icky,’”* Jill said this pensively while keeping eye contact.

“And how do you feel now?”

*“Scared, I think.”* Her eyes began to water with tears.

“Do you also feel a bit hurt? You have tears in your eyes?”

*“Maybe.”* Jill then described further her deep concern about her elderly mother and how she would be so *“upset if she dies.”* When asked what upset meant to her, she first said, *“Oh, upset like anyone is when their parent dies.”* When pressed to try and use more words specific to her, she paused. She said, *“I know that I have always lived as if my parents are always going to be here, sometimes that seems like it’s absolutely necessary—that I just can’t lose them, but other times, like right now, I know that I would feel sad if I didn’t have them, but I would keep on going.”* Her tears welled up as she said this, and rolled down her face, but she didn’t make any movement to wipe them away. The therapist commented that Jill had made a very clear statement describing her strong feelings without defaulting to her usual bodily focus. Jill replied, *“I know, and they seem true, and for once I am just saying them and not looking back in to see how my body is reacting as I say them.”*

The session was nearly over, but Jill recounted a few more times when she had been pre-occupied with her bodily sensations and missed out on experiences in life. Her voice was not harsh and she appeared to be reflecting with some compassion for herself.

In clinical case presentation #1 (**Box 1**), Jill’s history and symptoms appear to match the TAS-20 factor Difficulty Identifying Feelings (DIF). She uses bodily-based sensations as descriptors of her emotional state, such as that her heart is racing, or makes body movements to indicate the nature and experience of her feelings (e.g. fluttering her hands). She struggles to differentiate her bodily sensations from emotions, and frequently does not identify distinct feelings. Jill also persistently voices complaints of bodily-based symptoms that have been medical evaluated, with no pathology determined, such as her nausea and cardiac complaints. However, she does have a diagnosis of Irritable Bowel Syndrome (IBS) with no detectable organic pathology noted during screening evaluations.

Addressing Jill’s presentation from a psychological perspective, one relevant aspect is the difficulty that she has in using the signals from her body effectively regarding emotions—especially in relation to any organized storyline that could create narrative cohesion for her experience. Jill represents her mother as kind but minimally able to engage in any verbal consideration of her child’s affective or bodily state: only *“smoothing over”* Jill’s physical and social concerns with vague reassurance. Jill recalls the early memory of watching her mother tend to her sibling, while she sat nearby anxious with desire then deflated in resignation. Notably she remembers it in a manner that highlights her bodily experience. Her description of the family trip and her distress focuses on the physical experience, to the exclusion of the relational.

Jill’s descriptive style for her somatic experience, especially when she is trying to express an emotion, has been described by researchers as indicative of an inability to recognize how certain physical states are likely to indicate distinct feeling states (6). McDougall (74) has claimed that this form of speech is *“an act, rather than a symbolic means of communication of ideas or affect”* (p. 178). The therapist must recognize this style of communication as a signal reflecting the depth of the patient’s alarm, as she comprehends that the therapist is asking for words to describe feelings, yet she has no words available for these but only somatic experience. The therapist must not only tolerate the expressed alarm but also work to discern and comprehend the layers underneath the changing somatic symptoms expressed. It is often necessary for the therapist to utilize their countertransferential experience to discern the actual affective experience of the patient (5, 74).

It is worth noting that Jill has been diagnosed with IBS, a Functional Somatic Disorder (FSD) considered to result from a complex interaction between the individual and their environment (75). Psychodynamic considerations of such syndromes propose that patients use, “*so-called secondary attachment strategies (i.e., attachment deactivating and attachment hyperactivating strategies)”* [(75), p. 252], in reaction to experienced stressors. This decreases their ability to mentalize feeling states, thereby increasing the sense of both somatic and experiential distress, heightening attachment distress (28, 76). Jill expresses such by persistently wanting reassurance yet certain that her *“icky”* feelings will cause others important to her to leave her.

The Level of Emotional Awareness at which Jill operates is often between Levels 1 and 2 (15). Her focus on her bodily feelings is prominent, with a significant discordance between her above-average intelligence and her diminished vocabulary for feelings. Her level of emotional awareness is low because her affective states are not adequately attended to, in the context of her focus on the somatic. However, as she progressed through the period described in the vignette, she did have periods of Level 3 ability, as she was able to engage relationally with the therapist and to direct her attention more purposefully. When encouraged to seek different descriptive language rather than rely on habitual word choices, Jill moved further into Level 4, acknowledging blends of feelings. She was able to reference the somatic sense of *“icky”* to the expectation that the therapist would leave. Furthermore, she was able to verbalize awareness that she often imagines the negative, with her body as a focus, as *“nobody had asked how I was feeling really.”* She then verbalized a specific feeling state of sadness, regarding the potential loss of her mother, and noted that she was aware that she was then intentionally stating emotional feelings, and not referencing somatic experience, which she found encouraging.

Using the Three Process Model (24, 29, 31) to designate which processes related to emotional awareness might be disrupted the Affect Response Representation (ARR) process seems to be a significant limiting factor for her. Considering that *“the body is the first prior”* (71), it is notable, in Jill’s historical retelling of her life, that her sense that visceral reactions are sudden and portend something negative is prominent in each memory. The hierarchical processing in situations like this, suggests that there is a salient prior, with all new incoming interoceptive sensation interpreted according to this highly precise belief (prior), thus not allowing for alternative possibilities. There are disturbances in her attention regarding the social connections to which Jill has access. Therefore, incoming sensation from these connections that could lead to comforting emotional states is accorded very low precision, limiting her experiential response to her body only. This persists in Jill’s reaction in her group and is furthermore reified by her diminished effort to forage for new sensation, as evidenced by her speaking clearly in group and then not interacting further, later claiming that her body *“flared up”* and that was all she could think of at that moment. She also originally dismissed the therapist reaching toward her compassionately in the individual session—specifically, she looked away from her—again decreasing any new socially conveyed sensory information that might alter the high precision of her belief.

Box 2 **Clinical Case Presentation.**

Anna is a woman in her mid-50’s, unmarried with no children, who works as an executive in a large insurance company. She presented for therapy with complaints of *“things just aren’t going right.”*

Originally Anna reported she had no on-going illnesses, but when she was uncharacteristically late for a session she explained that she had *“one of those headaches.”* As her therapist probed for the symptoms of this recurring headache, it was clear that she probably suffered from migraines which had not been evaluated or treated. The patient was referred to a neurologist and was diagnosed with recurrent migraine. She has found relief from them with medication to preempt them, although she often does not take the medication in a timely manner. Anna paid close attention to her personal finances, was highly responsible with her bills, and didn’t buy herself very much beyond necessities. She remembered events in people’s lives very clearly, and she was always the first person in the waiting room before her weekly group session. She participated in a woman’s golf league every summer, keeping close watch on her score from week to week and very frustrated with her play if she does not score well.

While Anna is considered to be very kind and giving by those in her life, at work and by her friends, she has often felt as if she *“were on the outside looking in”* during social encounters. In therapy Anna often appears highly reserved, sitting stiffly in either group or individual sessions, often with her eyebrows knitted in thought and her mouth forming the shadow of a smile. When asked to describe her feelings, she usually first speaks in simple generalities. *“Meh”* is a common descriptor, which she claims is *“not good, not bad, and just not much of anything.”*

Anna’s mother figured prominently from the beginning in the telling of her history. She described her mother as chronically anxious, likely depressed, and emotionally unavailable to Anna from a young age. There were other significant familial issues. Her father abused alcohol and although he worked regularly, he would drink daily, often ending up on the couch, asleep and drunk. Her younger sibling, born 1 year after Anna, had behavioral disturbances from a young age and Anna was held responsible for managing this, whether they were in the home or out in the neighborhood. Anna’s memories of her mother centered on demands that she be compliant, that she control her sibling’s behavior and not ask questions.

Her mother resented any attempt by Anna to act independently and was constantly critical, when she might have felt pleasure or pride. Anna often references her mother’s severely disapproving look and being told in a harsh voice *“Who do you think you are?”* She commonly hears this phrase echoing in her head: when she is involved in her therapy; when she is describing an experience that was pleasurable; when she is describing anything she wants in her life; or when she is experiencing strong feelings in an intimate relational interaction.

If Anna announces that she can’t discern any feelings during an event that would seem to have associated affect, she has been encouraged to describe any feelings she is aware of in her body. At first, she would frown more deeply and then conclude she could feel no sensations beyond some tension in her shoulders. Over time, Anna has begun to describe feelings of anxiety and hurt. Associated with such feelings she also describes a *“feeling like I have this empty space in my chest and there is strong pressure over my shoulders pressing me down into the chair, almost like there are hands on my shoulders.”* She has verbalized how these sensations seem like a physical expression of the frequent harsh admonishments from her mother and her earlier necessary response to limit any expression of her emotions.

In a session a few weeks after Anna had helped to land a big contract at her office, an event that had been both exciting and pleasing to her, she was asked in her group session how she was feeling and replied, *“Oh, I am just back at ‘Meh.’ I don’t have any friends, don’t do anything but go to work and home, there is just nothing to my life.”*

A group member pleasantly said that she remembered a few sessions back Anna had been visibly pleased with her work and the compliments that she had received. Her current expression seemed discordant to the observing patient compared to that recent session. She described several qualities Anna had, that make her a good friend, and several interests she knew Anna had, such as politics and history. Other patients in the group concurred verbally, remembering interactions they had with her. Anna responded with a self-deprecating comment and went silent.

She was asked by her therapist to describe any bodily sensations that she felt. She described the space in her chest as before, and then she stopped. Encouraged to imagine if there was anything surrounding the space, she paused and then replied, *“It’s like there is rubber around it, just pushing and compressing everything into a smaller space inside.”* She made a crushing movement with her hands.

The therapist asked Anna how she felt regarding the other patient’s compliments to her. She said, *“I just hear her like she is just telling facts, I don’t feel anything.”*

Anna nodded, and then she appeared to tense more and her frown deepened.

*“What’s going on?”* asked the therapist.

“I don’t know, maybe like I am frozen still, like I just shouldn’t say or do anything?”

“Hmmm, what might happen if you did?”

“It’s like I would be found out.”

“Found out how?”

*“Like I’m not good enough.”* Anna said this with a severe frown, then plopped her hands into her lap and looked down.

The other patient spoke to Anna again, *“That sure would leave me feeling ‘meh’ Anna, but it does sound like you are kinda stuck in a ‘Who do you think you are?’ moment.”* Anna listened with a frown on her face and when asked what she felt she said, *“I don’t know what I feel, but it seems like there is just a force field around me. I can make an argument how each of my group members is wrong compared to what I see of my life, and could give them concrete examples. They just see snippets of it, not all of it.”*

Her therapist decided to take a tack that might elicit an experience of pleasurable sensation in Anna, which might then allow Anna to develop some awareness of associated affect, and then move on from there to other affect. She asked Anna if she ever reached out and felt the clothes in a store when she goes shopping *“to get a feel of the material?”*

*Anna grinned slightly and said she did*.

*“Can you describe the sensation that you might feel as you touch the clothes? Is there a sensation that you prefer?” the therapist asked*.

*Anna paused and said, “I do like the sensation of soft cotton, or really soft wool, but I don’t have money to spend on things that aren’t necessary, so I don’t usually go through the stores just looking at things.”* Anna was looking up, with a frown on her face, her hands pressed tightly against the arms of the chair.

“But you do have some sense of the sensation of the material and you do like the feeling of some materials more than others. You’re imagining that now, aren’t you—how these clothes feel good as you walk by and touch them?”

Anna smiled briefly and then returned to frowning. Another patient noticed the brief smile and asked her, *“What was that?”* Anna looked back at him and said, *“I* was *in a ‘Who do you think you are?’ place, and when she asked me to imagine that, how the clothes feel good, I then heard even more loudly in my feelings, “Don’t you even think of going there!”* She said this with a smile and then quickly quieted and frowned. Her therapist said, *“Wow, so not only ‘Who do you think you are?’ but you had better not move towards anything good or there will be hell to pay—but you can hear it so clearly, so how about you say it again, see what might happen?”* Anna repeated the phrase, *“Don’t you even think of going there!”* and was encouraged to do so several more times. Her group members looked on supportively, at times encouraging her verbally by accenting different parts of the sentence in between her statements. By the fifth time that she said it, she was speaking very directly to her therapist and loudly pronouncing the words, accenting the end of the sentence threateningly. At the end of the repetitions her posture was less tense, arms not braced, shoulders back, and her voice louder. She was smiling broadly.

“How does that tight feeling inside seem now?”

“It is different inside. It’s more like a container, softer plastic almost.”

“What might you feel?”

“Scared I think, it seems like that would be what I feel, I can feel it as tension, and I think that is scared.”

The group member who spoke to her originally said, *“It’s as if you are more open to us, as if your eyes are seeing us here in the room, and I can see your feelings in your eyes, so much more after you were so clear in your speaking.”* Anna then looked directly at the other patient and thanked her. Over the group she was interactive and commented on other’s experiences readily. At the end of the group her therapist returned to how, in repeating a phrase that seemingly told her not to go towards something that was good, Anna was more able to let go of that stance, and let herself be more open. Her therapist asked if she could further describe and express what she had experienced emotionally earlier. Anna paused for quite some time, and then said, *“It must have been fear because it is really easier now to “be on the inside of the group” and I can even imagine other things that I want to go toward. And that force field doesn’t seem to be here at all right now. I can say I feel kind of like ‘Meh’ plus, plus, now and that is actually pretty good.”*

Anna appears to experience the cognitive difficulty described by the TAS-20 facet, Externally Oriented Thinking (EOT). Her cognitive style matches Marty and de M’Uzan’s (4), *pensee operatoire*, as elaborated by Nemiah and Sifneos (6). Her thoughts are presented in a pragmatic manner, such as focusing more on her golf score than the pleasure of the game. She can be concrete in her interpretation, focused on the external, (only hearing *“facts”* when people are emotionally expressive towards her). *“Meh*” is her experiential description, describing a lack of affective vitality (8).

From a psychological perspective, Anna’s uber*-*focus on reality is underlain by a paucity of imagination, notably about herself. Krystal (5) notes the importance of the mother’s consistent presence in early life. The mother must seem to the infant to exist as an *“invisible holding environment,”* “just” long enough. This relational experience is described by Fotopoulou and Tsakiris (72) as if the caregiver’s body “*provide[s] sensory data that can plausibly be experienced by infants as their own.”* If this mother-infant body-to-body exchange changes too abruptly, and the mother is not available to the infant, the infant is suddenly left only with deprivation and unmet needs, and does not develop their ability to contextualize such. In other words, the ability to mentalize bodily sensations does not develop (72) and the patient is then unable to imagine that bodily sensations might reflect a need or a desire. This seems applicable to Anna, both regarding her presentation of her history (only 1 year between her and younger sibling—who is also noted as requiring more of the mother’s attention), and her description of her mother’s persistent critical responses to Anna’s expression of self-efficacy and emotional distance. Anna cannot be readily pleased with her accomplishments and has great difficulty in accepting other’s encouraging perspectives to seek what she needs or desires. She degrades each interaction by insisting she is not what the person says of her, she is *“just not good enough,”* thereby creating a barrier to her inner experience which (unconsciously) seems fraught with the danger of seeming abandonment.

In the household, Anna experienced her mother as persistently demanding that Anna take responsibility for minimizing disturbance in the house (e.g., limiting her active younger sibling). Experiencing ongoing admonishments as her mother’s efforts to limit her affective independence, Anna was presented with the option of repudiating fantasy or repudiating reality [(5); citing (77), p. 283]. Anna’s alexithymic presentation makes clear how this forced choice unfolded in her life; she had to repudiate fantasy, she cannot use her imagination, or express wants and desires, even to herself. She can’t even let herself imagine the pleasurable feeling of clothing she might buy and she immediately applies a possible reality-based reason as to why she doesn’t allow herself to shop, and therefore does not encounter any possible opportunity to imagine pleasure at such sensation.

The Level of Emotional Awareness (15) at which Anna generally operates is Level 2. Her over-focus on attending to reality, all diminish her emotional range. She does move towards Level 3 Emotional Awareness, expressing a muted experience of desire, when she is encouraged to imagine the feel of cloth against her skin. She then returns quickly to the place she knows well, vividly feeling her mother as present and that if she were even to imagine something pleasant to her senses she would be in great danger. So much so, that in Anna’s experience of the moment she spoke, as if a prohibition to herself, that she must *“not even think of going there.”* She has introjected her mother’s forbidding demand, leaving herself unable to formulate her own experience. She cannot just overcome, or let go of the fear, she must repeatedly, “inch by inch,” formulate her own experience. Yet, after interventions that specifically increase her body’s engagement and active support to take a more self-reflective stance, she is able to label the experienced bodily tension as fear, maintaining Level 3 emotional awareness.

With regard to the TPM (22, 24, 45), Anna has difficulty with the process of Affective Response Generation (ARG). The constriction of affect commonly seen in alexithymia is readily evident in her facial expression. However, as the session unfolds, deeper embodied roots regarding ARG become clear. As Anna describes her experience of the severely limited quality of her life, when an observant group member comments on the discordance between those statements and her very positive experience of Anna as a person who is kind and friendly, Anna first denigrates herself and then falls silent. Subsequently when asked about her bodily experience she can express the sensations, but she continues to remain withdrawn and without affective response, invoking the image of a seemingly physical *“force field”* that limits any view of herself as having positive qualities. She experiences again her mother’s statement, *“Who do you think you are?”* as a strong prior injunction against evaluating such interactions as available to her, i.e., not safe to explore. For example, during the interaction with her group member, Anna is unable to attend to novel stimuli and to be curious about what the other person is saying to her, sharply stopping the generation of positive affect and returning her to a self-deprecating and dismissive state regarding herself.^5^

Box 3 **Clinical Case Presentation.**

Susan is in her early 40’s, married, with a son. She has a college degree, and works as a librarian, after having tried several other professions. Recently she became a certified yoga instructor. She entered therapy complaining of feeling “*rudderless”* and as if she *“just couldn’t make a decision about anything”* and was stumbling around.

Her father was the more emotionally available of her parents, her mother prone to withdrawal. Susan described her mother with, *“her head in a book if she wasn’t cooking or cleaning.”* If Susan wanted her attention, it was hard to get. She describes her mother as *“self -involved, my whole life.”* Her father was congenial and pleasant. She remembers him taking her to softball practice regularly, following up on her school work, letting her wash his car, and taking her with him as he ran errands. Susan felt as if he took real interest in her and her activities.

A highly significant event in her early life was her father’s death when she was age 12. On the day of his death, Susan was home alone with her mother, her siblings were elsewhere. Her father was at an event in another city. The patient describes the event in the following exchange between the patient and her therapist.

This sense of waiting while something bad is about to happen, something over which she feels a sense of powerlessness, has been a prominent theme in Susan’s therapy. She has had periods when she was very quiet, with notable anhedonia. She complained that nothing made her happy, not her job, not her relationships. Group sessions were especially difficult for her, she felt as if no one was interested in what she had to say if they didn’t ask her directly, and she would remain quiet throughout. On leaving she would cry in her car all the way home. Arriving at her house she would inevitably start a fight with her husband, who would at times remain calm and at other times said that she was acting crazy and clearly her therapy wasn’t helping.

As an individual session began, Susan immediately began tensing up, and crying, harshly wiping the tears off her face, she said, *“I’m in that place again, that same damn place, all wound up tight and can’t feel anything, but my body is feeling something because my neck on the left side is starting to tighten.”* Her face contorts into a characteristic expression, with her mouth tightened, her pupils appearing almost fixed and not really seeing the therapist. She sobs in short bursts, holds her breath and has very deep horizontal furrows across her forehead. She says at these times, *“I can’t feel my forehead. It just feels like there is a blob over the front and top of my head, like I am just a blob.”*

Susan continued to cry and speak angrily about how she was *“back in that place”* and that she should *“not have to go there anymore.”* Stan (her husband) takes the brunt of it, *“last night I was just yelling again, I just lost it and kept on yelling about everything and nothing.”*

Her therapists asked Susan if she was *“willing to try something with me?”* She agreed. The therapist then asked *“What sensations do you feel in your body?”*

*Nothing really … something at my throat, tightness in my throat*.

Yeah? Tightness there, tightness how?

*I don’t know … like someone is trying to strangle me. Really tight at my throat and my neck, and I feel that blob over my forehead, that is all I can feel*.

Can you feel your feet on the floor?

(Moves her feet) *A little, I can feel the soles of my feet a little*.

Can you feel your bottom in the chair?

(Squirms a little) *Yeah, I can feel my bottom in the chair*.

*How about you feel what else you can feel as sensations, just sensations, in your body*.

(She begins to become tearful, her mouth grimacing, her breath is limited as she is holding her breath, body tense.) *I don’t know if I want to feel my body*.

No? It’s just sensations, what could happen?

*I don’t know … It just feels like I am this blob and I just want to run away. I can feel like my legs just want to run. And if I feel my body more there is just going to be more feelings like this and it is already really bad. I know that I have felt my body before and it even gets me out of this place, but I hate this place, everything seems so impossible*.

Ok. There is all that, the blob can take over if you let it. But how about you try to see what you can sense in your body, just see if anything comes to the fore?

(Silent for a bit) *Maybe something in my chest, maybe a tightness. Yes, a tightness here*. (Motioning over the middle of her chest.)

How is that tightness, is it keeping something in, or keeping something out?

*Keeping something in*.

*Yeah? How about you look at me as you try to feel any kind of sensation*. (Her eyes look at the therapist and then look away just over her shoulder, a habitual kind of eye movement for her.) *Are you here with me, or in there with you?* (The therapist points to her head.)

(She chuckles, and also tears come to her eyes*.) More in here with myself … The feeling in my throat is changing a little. A little less tight. And that feeling in my chest, it is moving a bit now. Like swirling*. (Motions with her hand*.) I can feel my shoulders and how tight they are. And my neck, you know how the left side of my neck always freezes up after my mother does her stuff. It feels like it is stiffening*. (Holding breath and squirming in her chair as she grimaces and cries.) *It is like no one is there, out there!!*

*Breathe. Just breathe*. (Susan lets out short sharp breaths.) *Breathe deeper, and try to keep your eyes on me*. (Therapist keeps direct eye contact and she looks back a bit more steadily but still looking away a bit, then she starts to weep and tears fall down her face)*. Can you see me?*

*Yes*.

Can you see my eyes, what color are my eyes?

*Brown? Yeah brown with a little lightness*.

What else is your body saying now?

*It is fighting to shut down but I can feel my legs a little more, and my hands, my shoulders*. (Moves them around.)

(Her breath is less constrained and she is looking out of her eyes more, not inside to herself.) *How is the blob right now?*

*More on my forehead, not all over my head. I can feel it as if it is just up here now, not all over my body as it was when I got here, just stuck there alone, like I am in a bubble and everything feels meaningless. Then I can feel like there is something at the back of my neck pulling all the muscles of my head*. (She puts her hand to the back of her neck as if pulling on a scarf, and she points to the prominent creases in her neck.) *It is as if all the muscles get so tight and I can’t think at all, about anything. The left side of my neck gets so tight, I can feel it now, but it is different than last night, it is softer now*.

What feeling would you describe that experience as, are there words for it?

*It’s scared, but no, no, it’s hurt too. A lot of hurt, and I can feel some anger. There are flashes of images moving through my mind*.

(She cries as she is looking at the therapist, her breath sometimes shallow, and then deepening. Her eyes look inside and then outside at the therapist.)

What are the images?

*Some have to do with that day that my dad died. Some are just colors, and then an image pops out*.

*seems not so scary, your breathing and eyes look less scared*, the therapist said compassionately.

*It isn’t, it’s more interesting now. I can look at it, and not just keep on fighting against the looking and pulling away*.

How did it happen again? That day, when your dad died. The phone call you always remember when you get upset and pull inside like this?

*The phone rang and my mom answered it. The look on her face just changed so quickly. She dropped the phone back so quickly and I said, “What happened?” She stared straight ahead and said, “Dad had a heart attack, they are taking him to the hospital.” I yelled, “What, what?” But she just said “They said to wait to hear something.” She just sat there staring straight ahead, we both just sat at the table for so long. Then the phone rang again. It was ringing and ringing, she wouldn’t pick it up. I yelled at her to pick up the phone!! She wouldn’t. Finally I picked it up and Grandpa said, “Your dad died, they couldn’t save him.” My mom still wouldn’t take the phone! She just said “what are we going to do” over and over again. I remember that I was on the floor crying and pounding and she said, “Stop that, just stop that.” She sat there at the table, just saying over and over, “What are we going to do?” but she wouldn’t talk to me*.

So you just shut down. But you have images in your mind today?

*Now I can see the phone ringing on the counter, and me screaming at her to answer the phone, and seeing myself answer it and then nothing. I couldn’t see or hear anything after that, seems like for so long. It was like after that I had to be squashed, just squashed, nothing could go out*.

*And nothing could come in*.

(Cries heavily.) *Yeah. I didn’t know what to do, how to be, I just had to put my head down and read in school, but I didn’t learn anything. I went to school but I just went through the motions. I was so scared all the time, and just did exactly as I was told. I studied and gave it back to them how they wanted it but didn’t know what I wanted. I did everything by rote, I didn’t know what I was doing, and there was no one to ask. And then that day, she didn’t say anything for all that time. It just seems to get like that kind of quiet in my head and then I get all bound up and I just get all tight and fuss and keep people out, like I am squashed again in all this silence, and I won’t let anyone in. Do you remember when I came to therapy and I didn’t know what I wanted to do, I was just wandering. I thought I would be a teacher, or maybe a doctor, or maybe just stay at home and take care of our son*.

*Yes, I do, and I also remember how you would find something you really liked then suddenly leave it*.

*It was like I couldn’t let anything just “be,” I would be lost in this silence. I couldn’t make any choices, and if I did make a choice when it was good I would have to just quickly leave it. But I can see now that I was just waiting, waiting for something to pass, or something to happen I don’t know, I couldn’t stand the feeling of waiting but the idea of moving towards something was just overwhelming*.

Without your dad with you?

It must have been, I know that my feelings seemed so muted for such a long time. I couldn’t feel anything a lot of the time, or I knew there was something but didn’t know what. At least last night when I got in a fight with Stan, at least then I could say, wait a minute, and I told Stan that I was just going to take a walk, I would be back. I took a walk down the street, smelled the fall air, and came back, and apologized for the yelling. Stan said ok, and he even said that he had “fallen into it too.”

“Today you were able to come out of the squashed place and tell me what was happening inside. And even have some images to see, not just silence. And the blob that seems to take over your thinking and feelings isn’t doing that now. Your eyes are clearly looking at me. You were able to move through the feelings, even call them something, and that tightness in your muscles seems a lot less, the lines in your forehead are even less. How is that for you?”

“It is good. I do feel movement in my muscles, my breathing, as if I have some say in things and can say it, and even feel too. Breathing does help. I know I say it in my yoga classes a lot, sometimes I hear it too, even!”

The TAS-20 factor that fits Susan’s symptoms is Difficulty Describing Feelings (DDF). She initially describes her emotional experience only as a sense that she “*just a blob.”* There are long periods during which she experiences severely restricted affect, interspersed with intense sudden periods of dysregulated action, which have been noted to occur in alexithymic patients. For example, Taylor, Bagby, and Parker (8) state that when such outbursts occur, if patients are asked what they feel, they are often unable to link such activity with feelings. Affect is expressed only as action. In the midst of strong feeling states, Susan can comment that agitated activity is likely related to feeling experience, but she cannot label what the feelings might be, even when there are clear antecedents that can be tied to memories of earlier events.

From a psychological perspective, her father’s death and the events she remembers surrounding that event, echoed through Susan’s life in many different ways. Susan describes this affective regression in clear terms: she *“did everything by rote*”; she used action instead of words to describe her distress; and *“I would just be lost in the silence.”* She could not gain conscious awareness of her feelings due to this anhedonia, which was self-reinforcing. Significant anhedonia associated with alexithymia is noted by Krystal (5) as likely to result from a trauma occurring later in life and is a *“regression of affect,”* resulting in a severe disruption of subjective experience due to the “*dedifferentiation, deverbalization, and desomatization of affects”* [(9), p. 1010]. Susan describes every aspect of this experientially: dedifferentiation—she feels as if *“I am just a blob”* unable to describe individual feelings; deverbalization—*“I was just lost in the silence”*; desomatization—when asked what sensations she feels in her body she claims *“Nothing really.”* Susan’s Level of Emotional Awareness (15) appears to have variability functionally. She can be stuck at Level 1, where she is only aware of the tension in her body and her anhedonia, and is quiet in her group only to cry in the car, without identifying any associated feelings. At times, she also reaches Level 3 and then Level 4, expressing awareness of feeling angry, scared, and hurt, which are blends of feelings, and blends and blends of feelings.

Regarding the Three Process Model (TPM) (22, 24, 73), Susan struggles with allowing affective experience into conscious awareness (CA). She initially can only describe her experience with somatic description and she readily berates herself, *“I’m in that place again, that same damn place, all wound up tight and can’t feel anything.”* While in conversation, Susan can readily remember the events surrounding her father’s death and will then describe how she experienced her mother’s refusal to answer the phone and her leaving Susan to be told the news. Susan repeatedly re-enacts this event with her family and outside the group (periods of waiting silently and then behavioral disruption)—without placing her state or the actions she undertakes within the context of this event—although she ultimately does so in the safety of the individual therapy session described. This again highlights the necessity of the therapeutic relationship as an anchoring presence for patients who are in the throes of strong feelings, in order to allow feelings into awareness and pursue emotional regulation strategies (78). We will consider in the next section how important this process is for the patient, to encourage attentional shifts and allow for deepening mentalization processes to support change in the patient.

## Therapeutic Considerations

There are, of course, many psychotherapeutic treatments for the disturbances in alexithymia. My specific focus in this paper, however, has been on how comprehending of recent advances in the understanding of the neurobiological processes, when applied within the context of the therapeutic relationship, can enhance the clinician’s treatment of the alexithymic patient, within any psychotherapeutic protocol. I hope that clinicians will be interested in these elements and adapt them appropriately, in accordance with their personal theoretical standpoint and psychotherapeutic treatment process.

The core of the insights from the theory presented in this paper is that the relative precision of prior beliefs or sensory evidence is vital to the accuracy of the interoceptive inference processes that are continuously unfolding in all individuals at all times. It is my contention that patients with alexithymia function with suboptimal interoceptive inference processes, because their priors (predictions/beliefs) are resistant to change. These patients’ attention to their current sensations (and to use these to refine their beliefs) is hampered by their heavily entrenched, habitual interoceptive inference processes. There are distinct reasons for this, unique to each individual and developing from infancy onward.

While we may not be privy to the direct causes of our patients’ suboptimal predictions that are so resistant to change, there are entry points available within the therapeutic relationship that can help the patient to increase their sampling of the evidence currently available to their senses, and thus improve their inference processes, and increase their emotional awareness. One important purpose in this paper is to propose that improving the flexibility of patients’ attentional control is a key entry point.

Attention can be regarded as a volitional *“direction of consciousness”* (79, 80). For our purposes, we start with the premise that the Bayesian brain is attempting to *“optimize the evidence for its model of the world”* [(81), p. 16]. Within this framework, attention is activity that infers the precision of the sensory input relative to the predicted state of the world (i.e. to the prior belief). Feldman and Friston (81) also note that attention highlights information from a certain source and that this will increase the precision of that sensory information relative to the top-down prior. To this end, directed attention to sensation vs prior belief will affect the weight of prediction errors as they move up the hierarchy, thus affecting the precision of that stream of sensory stimuli relative to the prior prediction (81).^6^

Patients’ models of the world rest on the relative precision that their prior beliefs have relative to the precision of incoming sensation. As described above, in alexithymia, there are various ways in which the precision of over-learned priors may gain excessive strength throughout development, which is later expressed in adulthood. Linson et al. (82), for example, describe instances in which an *“over-fitting”* of sensations occurs, with over-weighted (over-precise) interoceptive sensory experience driving the predicted state, such as in somatic preoccupation (as for Jill, in case study #1). By contrast, in states dominated by precise predictions that stem from prior trauma, there is likely an *“under-fitting”* of sensory information relative to the prediction (70), with a lack of sensory sampling resulting in low precision of sensory information and a predicted state that is highly uncoupled from the actual environment [as for Susan, in case study #3 (**Box 3**)]. Within each of these examples, it is the flexible application of attention between interoceptive and exteroceptive signals and between the sensory information and the patient’s habitual priors that can generate changes in precision and allow updating. This flexibility is a skill vital to develop in patients, supporting active changes in the generative models of interoceptive inference, and allowing emotional experience to reach awareness. Important aspects of the therapeutic relationship are essential to provide a much needed, felt sense of safety in our patients, in order to allow this flexible deployment of attention. We will now consider application of these ideas and principles utilizing the case studies described above.

Addressing Jill’s (case #1) symptomatic presentation from a predictive processing perspective, her persistent attention to bodily sensations (as well as her interpretation of them) reinforces the precision (the salience) of a high level prior for illness. It also diminishes Jill’s ability to infer and identify feeling states (79, 81). Her attention being largely directed toward priors that are illness-related beliefs has the effect of increasing the precision of these priors, thereby further magnifying Jill’s attention to her body (that she expects to exhibit symptoms of illness). An initially weak (imprecise) interoceptive signal indicating any kind of illness in her body will therefore become more precise (by the attention she focuses on it), thereby increasing the salience (precision) of the original prior prediction for illness. This results in a “*self-perpetuating cycle*” (83), where worrying about potential illnesses increases the precision of the associated prior and thus the person’s belief that they have an illness becomes stronger. Van den Bergh et al. (83), propose that *“symptoms are experienced when the generative model with the lowest overall prediction error represents an interoceptive event with an abnormal (typically disease) cause”* [(83), p. 194]. Conversely, interoceptive signals that may simply signify affect are ignored, or are misinterpreted as sickness. In Jill’s case, even with negative objective test results, somatic sensations are insistently expressed as a subjective belief that she is still probably sick.

Meditative practices could support Jill’s efforts at de-centering from her bodily experience. They promote a *“disciplined attention”* (84), increasing compassionate and non-judgmental approach to bodily sensation that can support stability in the homeostatic range of the individual. This could be important for Jill, who experiences any change in physiological sensation as threatening. Meditation enhances an individual’s ability to manage such processes, thereby decreasing the expected position (the prior) that they are powerless to control the efforts of their mind or, at a more direct level, their thinking (as Jill insisted at first). Such changes in modulation of interceptive information, and subsequently in self-experience, can also affect the compassion and regard the individual holds for themselves and that they are able to accept from others—important features of change (85). Jill could eventually speak more kindly about her sadness, regarding the experiences she had missed while preoccupied with her bodily experience, expressing an increased level of emotional awareness.

The purposeful direction of attention (with relational support) to physical sensation described in Susan’s case (case #3) supports epistemic foraging. The purposeful shifting of her attention from exteroceptive to interoceptive sensation greatly increases the flow of sensory information, allowing her to use more personally relevant and descriptive language regarding her somatic experience. There is increased conscious access to old priors that she is reacting to, with expressed insight into those priors, and increasing levels of emotional awareness. As Smith (24) asserts, this attentional process promotes a reappraisal of her situation (such as is intended in therapies such as Cognitive Behavioral Therapy), which affords Susan the opportunity to explore new priors or alternative explanations for these (now attended) bodily sensations, producing *“a different and more adaptive affective response”* [(24), p. 45]. Furthermore, this could lead to such old memories (or habitual priors) becoming linked predictively with the new and healthier affective responses, altogether changing how they are stored and recalled (24, 29, 45).

The focus on the interoceptive sensation of breathing, and compassionate body interest promoted by her yoga practice, invokes a new experience of self-care for Susan. It alters the old prior, established with her mother, that there is no available care for her, which stimulates her to dismiss all interoceptive sensations. She reports that yoga has been a meaningful place for her to gain a sense of agency, expressed as her purposeful breath focus, which reminds her that she has *“some say in things.”* She describes using such self-calming practices at home, albeit sometimes after she has been too reactive to her prior beliefs regarding others, such as her husband. This increase in agency is accompanied by increasing emotional awareness, with blends of feelings being verbalized. As this shift in bodily experience allows a loosening of the hold that the generative model of an absent mother has on her physical reactions, Susan can now also describe a generalization of such change in cognitions.

The effect of highly precise priors on cognitive style and bodily state are evidenced in Anna’s presentation [clinical case #2 (**Box 2**)]. Her strong prior for interpreting most interactions as involving her mother’s exhortations (the strongly prohibitive *“who do you think you are?”*) limits any attention to interoceptive sensation, keeping her attentional focus on the external, which greatly limits awareness of her emotional state and that of others. Her limited seeking of new experiences, of any kind that might instigate emotional response, does not allow for the mentalization of affect, leaving her only with the sense that life is *“not good, not bad,”* or with the vague descriptor *“meh,”* with which to express her feeling state.

Nevertheless, as Anna responds to the invitation to loudly express the prohibition that she constantly experiences, the attention this affords to her bodily sensations increases the precision of these sensations relative to the precision of the habitual prior. As a result, alternative priors that she would not previously have entertained, can now “get a hearing,” for example, that it is safe for her to actively move and express herself loudly, without the expected rejection. Having alternative priors to select between induces conscious awareness and enables interoception to be mentalized; thereby providing therapeutic access (51). Anna’s group members support her expression of her own power. They are encouraging as they describe meaningful changes in her physical presentation and emotional availability, thus further increasing attention to (i.e. the precision of) this new conscious prior, while challenging her habitual reactions as no longer fitting. As prediction errors are resolved between this new high level prior for safety, which now matches the low level sensory data in the present moment, Anna steadily calms down and even smiles. She concludes with an increase in emotional awareness, elevating her experienced sense of feelings from *“meh”* to *“meh, plus, plus,”* and that is pleasing to her.

Importantly, as the therapist endeavors to help the patient direct attention to current experience, in order to affect change in habitual priors that are overly precise, increasing uncertainty is generated. This therapeutic activity therefore requires vital experiential anchoring over time, actively promoted within the therapeutic relationship. This is provided by readily available aspects in the relationship—such as the authentic and genuine qualities of the therapist, which can be described as elements of the *“real relationship*” (78). Morgan et al. (86) notes that the interactional elements based in the real relationship, available to the patient, increases their experienced sense of safety. Thus, the real relationship allows *“a space for departure from past experiences with other people”* [(86), p. 327]. For the alexithymic patient, a therapist’s honest expression of curiosity, emotional availability, and reflective stance are powerful resources with which to change overlearned suboptimal priors. The therapist’s use of emotionally-valenced language also increases the patient’s lexicon for such language. The real presence of the therapist is fundamental to patients’ efforts to effect change in their process of living, with themselves and others, regardless of which element is disturbed in the initiation of an emotional episode (or in awareness of emotional experience), or what habitual reaction stifles emotional expression.

## Conclusion

The disturbance in emotional awareness in alexithymia can be described in many ways. Ongoing research is throwing light on the processes that underpin this disruption and likely means to remediate the difficulties of alexithymic patients. In this article I have described how our patients’ unique presentations of alexithymia, and their limitations in emotional expression and awareness, are the result of the hierarchical interoceptive inference processes, by which the brain actively attempts to discern the cause of incoming sensory stimuli. In alexithymia, this process takes place under the ongoing influence of: strongly held suboptimal prior beliefs; dysfunctional attentional control; and diminished ability to seek alternative evidence (known as epistemic foraging). In the light of recent innovations in neuroscientific theory and its application to psychotherapy, I have argued in this article for the vital importance of enhancing our alexithymic patients’ ability to flexibly switch their attention between: their incoming interoceptive sensations; exteroceptive sensory signals from the world, in the current moment (i.e. the context); and their own habitual and deeply held prior beliefs about the causes or meanings of sensations. Among other therapeutic benefits, the patient will exhibit increased emotional awareness, affective expression, and mentalization, and improve their relational experiences on many levels. I hope that this characterization, and the recommendations that follow from it, will be of values to therapists of all persuasions, in their attempts to alleviate the difficulties that bring such patients to us.

## Data Availability Statement

The datasets for this manuscript are not publicly available because: The information was attained in psychotherapy sessions that are confidential. The author received express written consent from each individual(s) regarding the clinical material that is disguised as to the individual’s identity.

## Ethics Statement

Ethical approval was not provided for this study on human participants because the paper involves case studies of patients in private treatment. Written, informed consent was gained from each patient, and all identifiable material was changed to protect their identity.

## Author Contributions

PD contributed solely to this work.

## Conflict of Interest

The author declares that the research was conducted in the absence of any commercial or financial relationships that could be construed as a potential conflict of interest.

## Footnotes

1 Within this paper, a distinction is made between the terms *emotion* and *feelings*. This is in accord with Damasio’s assertion that emotions are embodied experiences, while feelings are the awareness of having emotions (3). The term *affect* is used here to include both emotion and feeling (6).

2 Brain regions identified as DMN include medial prefrontal cortex, posterior cingulate cortex, ventrolateral prefrontal cortex, lateral temporal lobe 37.

3 While most interoceptive activity occurs outside of conscious awareness, some sensation rises above the brainstem, such that most people are more or less aware of sensation from at least four interoceptive channels which are: cardiovascular; respiratory; visceral (the gut); and urogenital. The research findings that have looked for a relationship between sensitivity to, or awareness of, interoceptive signals and alexithymia unfortunately have failed to reach a consensus. This is due to significant methodological differences and different measurement instruments. The interested reader is directed to the literature for more detail, for example: (13, 16, 54–56).

4 For the purposes of this paper active inference will be considered synonymous with interoceptive inference.

5 One could make the case for fear being generated and causative in this interaction. However, this would be a confound within the Affective Generation Response process, as fear is an affect and is also the most common denominator in any situation in which an individual does not feel safe.

6 Such activity as described here actually occurs within the brain through changing levels of cellular activity, with individual cells and networks of the brain activated or inhibited.
